# Association of the *FAM46A* Gene VNTRs and *BAG6* rs3117582 SNP with Non Small Cell Lung Cancer (NSCLC) in Croatian and Norwegian Populations

**DOI:** 10.1371/journal.pone.0122651

**Published:** 2015-04-17

**Authors:** Godfrey Essien Etokebe, Shanbeh Zienolddiny, Zeljko Kupanovac, Morten Enersen, Sanja Balen, Veljko Flego, Ljiljana Bulat-Kardum, Anđelka Radojčić-Badovinac, Vidar Skaug, Per Bakke, Aage Haugen, Zlatko Dembic

**Affiliations:** 1 Institute for Oral Biology, Faculty of Dentistry, University of Oslo, Oslo, Norway; 2 Section of Pulmology, Department of Internal Medicine, Clinical Hospital Center, University of Rijeka, Rijeka, Croatia; 3 Department of Chemical and Biological Working Environment, National Institute of Occupational Health, Oslo, Norway; 4 Institute for Transfusion Medicine, Clinical Hospital Center, University of Rijeka, Rijeka, Croatia; 5 Department of Clinical Sciences, University of Bergen, Bergen, Norway; 6 Department for Biology and Medical Genetics, Faculty of Medicine, University of Rijeka, Rijeka, Croatia; Duke Cancer Institute, UNITED STATES

## Abstract

We analyzed for associations between a variable number of tandem repeat (VNTR) polymorphism in the Family with sequence similarity 46, member A (*FAM46A*) gene and a single nucleotide polymorphism (rs3117582) in the BCL2-Associated Athanogene 6 (*BAG6*) with non small cell lung cancer in Croatian and Norwegian subjects. A total of 503 (262 Croatian and 241Norwegian) non small cell lung cancer patients and 897 controls (568 Croatian and 329 Norwegian) were analyzed. We found that the frequency of allele b (three VNTR repeats) of *FAM46A* gene was significantly increased in the patients compared to the healthy controls in the Croatian and the combined Croatian and Norwegian subjects. Genotype frequencies of cd (four and five VNTR repeats) and cc (four VNTR repeats homozygote) of the *FAM46A* gene were significantly decreased in the patients compared to the healthy controls in the Croatian and Norwegian subjects, respectively. Logistic regression analyses revealed *FAM46A* genotype cc to be an independent predictive factor for non small cell lung cancer risk in the Norwegian subjects after adjustment for age, gender and smoking status. This is the first study to suggest an association between the *FAM46A* gene VNTR polymorphisms and non small cell lung cancer. We found also that *BAG6* rs3117582 SNP was associated with non small cell lung cancer in the Norwegian subjects and the combined Croatian-Norwegian subjects corroborating the earlier finding that *BAG6* rs3117582 SNP was associated with lung cancer in Europeans. Logistic regression analyses revealed that genotypes and alleles of *BAG6* were independent predictive factor for non small cell lung cancer risk in the Norwegian and combined Croatian-Norwegian subjects, after adjustment for age and gender.

## Introduction

Lung cancer is the most predominant cause of cancer death globally [1]. Through epidemiological studies many environmental risk factors have been established for lung cancer including smoking, air pollution and industrial substances [2]. Although tobacco smoking is the major risk factor, genetic factors also affect lung cancer susceptibility [3–5]. Direct evidence for genetic predisposition to lung cancer is highlighted by several genome wide association studies (GWAS) that has been done [6–11].

Most of the genetic association reports studying lung cancer use single nucleotide polymorphisms (SNPs) as markers. A genomic variant that is understudied is the variable number of tandem repeats (VNTR) probably due to VNTR complexity and the challenges in assaying them. These limitations do not favor the discovery of novel VNTRs as potential predictive and prognostic factors in lung cancer etiology. Predictive and prognostic factors are important in the diagnosis and treatment of lung cancer [12–14]. The positive long term economic impact of robustly testing for predictive factors cannot be underestimated. This enhances the quality of medical care by significantly reducing false positives or negatives that may impact negatively on the treatment outcome. For example, robust testing for epidermal growth factor receptor (*EGFR*) gene mutational status in non small cell lung cancer (NSCLC) patients has defined this gene as an important predictive and prognostic factor in NSCLC diagnosis and treatment. This has also allowed for the therapeutic targeting of this genetic locus in NSCLC treatment [15;16].

VNTRs can modulate many biological processes such as gene transcription and protein function. They may also be responsible for many disorders in humans that include unstable (genetic) repeat expansions [17;18]. There are few reports on the association between VNTRs and lung cancer susceptibility in case-control studies. These include VNTRs in the *H-ras* gene [19;20], interleukin-1 receptor antagonist gene (IL1RN *2) [21;22] and Mitogen-activated protein kinase 2 gene (*MAPKAPK2*) [23].

The Family-with-sequence-similarity 46, member A (*FAM46A*) gene [24] is located at chromosome 6.14.1. It harbors a VNTR within its coding sequence in exon 2. This VNTR may vary from two to seven repeats per chromosome [25;26]. The *FAM46A* polypeptide chain also contains the Domain of unknown function 1693 (DUF1693) [27] and as such no biological role has been assigned to the *FAM46A* gene as of date [28;29]. *FAM46A* protein interacts with the *BCL2*-Associated Athanogene 6 (*BAG6*) protein [28] which has been reported to modify risk of lung cancer [30]. The *BAG6* gene is located on chromosome 6p21.3 and regulated apoptosis and HSP70 [31]. *FAM46A* protein also interacts with the zinc finger, FYVE domain-containing 9 (*ZFYVE9*) protein [28] which is involved in *TGF-β* signaling.

We previously reported that the mouse homologue of the *Fam46a* gene is expressed in developing tooth buds. Due to its nuclear localization and interaction with the human transcription factor, *ZFYVE9* protein, we suggested that the *FAM46A* protein might be involved in cellular proliferation [32]. In addition, we have recently reported that the *FAM46A* gene VNTR is associated with increased risk of tuberculosis and osteoarthritis [33;34]. Data suggest that patients with tuberculosis are associated with increased lung cancer [35]. Based on these facts, we hypothesized that the *FAM46A* gene may be involved in lung cancer and that this involvement may be through variations in the length of the *FAM46A* VNTR. In addition, we analyzed for the association of a *BAG6* SNP to validate its previous reported association with lung cancer [30]. The associations were investigated in two different European populations.

## Materials and Methods

### Ethics Statement

The study was approved by the Medical ethics committees of the University Hospital, University of Rijeka, Croatia, and Regional Committees for Medical and Health Research Ethics, Oslo, Norway. Written consents were obtained from all participants.

### Subjects

The number of participants, sex and age distribution of the subjects are described in Table 1 for the both the Croatian and Norwegian subjects, respectively. Blood samples were collected at the Clinical Institute for Transfusion Medicine, University Hospital Center Rijeka, Rijeka, Croatia for the Croatian subjects and at National Institute of Occupational Health, Oslo, Norway for the Norwegian subjects. Ethnicity of participants was established by patient or healthy individual interview and by consulting the admission documentation at the hospitals. Some clinical information for particularly subjects was lacking and as such, not all subjects were included in the characteristics estimations in Table 1. Subjects were not matched for possible confounding factors such as age, gender, and smoking status. Not all patients and controls were typed for the two markers due to lack of particular samples.

**Table 1:** Characteristics of non small cell lung cancer (NSCLC) patients and normal (healthy) controls.

Subjects	Characteristic	Patients	Controls	p-value
Croatians	n^a^	215	471	
	Average age (males and females)	66.12 (±10.39)	42.83 (± 10.84)	<0.0001
	Age range (males and females)	31–92	19–73	
	n^f^	57	189	
	Average age (females)	66.93 (±10.99)	45.12 (±10.99)	<0.0001
	Age range (females)	47–85	21–68	
	n^m^	158	282	
	Average age (males)	65.82 (±10.18)	41 (±11.55)	<0.0001
	Age range (males)	31–92	19–73	
	Smoking status	never: 37	never: na	
		ever: 225	ever: na	
	Histology	Adenocarcinoma: 67		
		Squamous carcinoma: 113		
		Large cell carcinoma: 3		
		undetermined NSCLC: 79		
Norwegians	n^a^	238	292	
	Average age (males and females)	64.24 (±10.22)	59.66 (±10.34)	<0.0001
	Age range (males and females)	25–82	40–88	
	n^f^	70	69	
	Average age (females)	64.04 (±10.42)	61.63 (±10.98)	0.19
	Age range (females)	36–81	40–83	
	n^m^	177	223	
	Average age (males)	64.45 (±10.16)	59.06 (±10.08)	<0.0001
	Age range (males)	25–71	40–88	
	Smoking status	never: 17	never: 0	<0.0001
		ever: 229	ever: 292	
	Histology	Adenocarcinoma: 148		
		Squamous carcinoma: 98		
		Large cell carcinoma: 34		
Pooled Croatians and Norwegians	n^a^	453	760	
	Average age (males and females)	65.13 (±10.16)	49.29 (±10.16)	<0.0001
	Age range (males and females)	31–92	19–88	
	n^f^	124	258	
	Average age (females)	65.16 (±10.16)	49.53 (±10.16)	<0.0001
	Age range (females)	36–85	21–83	
	n^m^	329	502	
	Average age (males)	65.12 (±10.19)	49.17 (±14.07)	<0.0001
	Age range (males)	25–92	19–88	
	Smoking status	never: 54	never: na	
		ever: 454	ever: na	
	Histology	Adenocarcinoma: 215		
		Squamous carcinoma:211		
		Large cell carcinoma: 37		
		undetermined NSCLS: 79		

n^a^, male and female subjects;

n^f^, females subjects;

n^m^, males subjects;

na, not available.

### Genomic DNA extraction

Genomic DNA from NSCLC patients and healthy controls was extracted from whole blood as described previously [36–38]. In brief, 200 μl of whole blood was mixed with 400 μl of sucrose buffer (0.32 M sucrose, 10 mM Tris–HCl, pH 7.5, 5 mM MgCl2, 1%, v/v, Triton X-100) and incubated for 1 min at room temperature. To collect white cell nuclei, samples were centrifuged 2 min at 5,000g. Precipitated nuclei were washed twice with 800 μl of sucrose buffer and centrifuged (2 min at 5,000g). After the second wash, the nuclei were re-suspended in 400 μl DNAzol (Invitrogen Corporation, Carlsbad, CA, USA) and incubated at room temperature for 5 min. Genomic DNA was precipitated with 200 μl of 100% ethanol and collected by centrifugation (2 min at 5,000g). The precipitate was washed with 1 ml of 75% ethanol and centrifuged for 1 min at 5,000g, twice. Genomic DNA was re-suspended in Tris-EDTA buffer solution (10 mM Tris-HCl, 1 mM disodium EDTA, pH 8.0; Sigma-Aldrich Chemie Gmbh, Munich, Germany).

### Genotyping by DNA-sequencing Capillary Electrophoresis

DNA fragments of 647 base pairs (bp) in length encoding the FAM46A gene were amplified from human genomic DNA by using a FAM-labeled forward primer, designated Gfam_VF (5′-AGGGTACTTCGCCATGTCTG-3′), in combination with an unlabeled reverse primer, designated GEX_R (5′-CTCGTGATGGCCACAGATT-3′), by PCR as previously described [33;34]. The 25 μL total volume PCR mixtures contained the following: 25 ng of genomic DNA, 0.2 μm each of the specific primers, and 1x Paq5000 Hotstart PCR master mix (Agilent Technologies, Inc., CA, USA). PCR was performed in a Peltier Thermal cycler (MJ Research, Massachusetts, USA). The Paq5000 polymerase was activated by an initial step at 95°C lasting 2 min, followed by 35 cycles of denaturing, annealing, and extension steps at 95°C for 20 s, 65°C for 20 s, and 72°C for 30 s, respectively, followed by a final extension step at 72°C for 5 min. Amplicons were resolved by 1% ethidium bromide-stained agarose gel electrophoresis and visualized by the Geldoc imaging system (Bio-Rad, Hercules, CA, USA). Amplicons (0.5 μl) were mixed with 0.5 μl GeneScan 1200 LIZ Size Standard (Life Technologies, NY, USA) and loaded onto a 3730 DNA Analyzer (Life Technologies, NY, USA) for allele separation. Separated alleles were analyzed by the Genemapper software (Life Technologies, NY, USA). Allele (VNTR) identity was confirmed by sequencing directly PCR amplicons from samples that were genotyped as various homozygotes (two each). To further confirm the genotyping results, 10% of the samples were re-genotyped with 100% concordance. Also, randomly selected samples that were genotyped as heterozygotes were sub-cloned into TOPO Zero Blunt Sequencing plasmids (Life Technologies, NY, USA) prior to sequencing. Sequencing reaction was performed using the BigDye chemistry 3.1 (Life Technologies, NY, USA) with forward and reverse primers GVF (5′-AGGGTACTTCGCCATGTCTG-3′) and GEX_R (5′-CTCGTGATGGCCACAGATT-3′), respectively and resolved by the ABI 3730 DNA analyzer (Life Technologies, NY, USA).

### Genotyping of *BAG6* rs3117582 Single Nucleotide Polymorphism (SNP)

BCL2-Associated Athanogene 6 (*BAG6*) gene rs3117582 SNP was assessed by probe-based real-time PCR assays as described by the Kits manufacturer (Life Technologies, NY, USA) in our NSCLC patient and control groups, respectively. Stratagene MX3005 real-time PCR cycler was applied (Agilent Technologies, Santa Clara, CA, USA) for temperature cycling and signal quantification.

### Statistical analysis

The differences in the distribution of categorical variables, including demographic characteristics, selected variables, allelic and genotypic frequencies were analyzed by the chi-square (Fisher two tailed) method using the 2-way Contingency Table Analysis (available at http://statpages.org/ctab2x2.html and http://research.microsoft.com/en-us/um/redmond/projects/mscompbio/fisherexacttest/) or t-test (http://www.graphpad.com/quickcalcs/ttest1/?Format=SD) between patients and controls. The Hardy-Weinberg analysis was done using the Arlequin software version 3.5 (Genetics and Biometry Laboratory, University of Geneva, Geneva, Switzerland), which showed that the *FAM46A* VNTRs and the *BAG6* genotypes were in Hardy-Weinberg equilibrium (HWE). A statistically significant difference was defined when p was ≤0.05. The associations between the *FAM46A* VNTRs and *BAG6* rs3117582 SNP polymorphisms and non small cell lung cancer risk were estimated by computing the odds ratios (ORs) and their 95% confidence intervals (CIs). We performed logistic regression analysis to evaluate the influence of age, gender and smoking status as confounders for the association of *FAM46A* VNTRs and *BAG6* rs3117582SNP, respectively, with non small cell lung cancers in the Croatian and Norwegian subjects. Smoking status could not be included as a cofounder when we analyzed for the association of these genetic elements with non small cell lung cancer in the Croatian subjects and the combined Croatian-Norwegian subjects because information on the smoking status of the Croatian healthy control group was not available. Logistic regression analyses were carried out using SPSS (version 21; SPSS Inc., Chicago, IL, USA) and a p-value of ≤ 0.05 was set as the criterion for statistical significance.

## Results

### Allelic and genotypic frequencies of the *FAM46A* gene VNTR

The frequency of VNTR genotypes and alleles for the *FAM46A* gene in the patients and healthy controls are listed in Table 2 and 3. We found twenty genotypes comprising of six alleles for the *FAM46A* gene with different frequencies in the two populations. Therefore, in further analyses of genotypes we chose to analyze results separately for each population, in addition to performing combined analyses. Analysis of the allelic frequencies, however, showed that the VNTR with 5 repeats (d allele in Table 3) was the most frequent allele with a similar frequency in healthy controls in both the Croatian (42%) and Norwegian (40%) populations. We chose, therefore, d allele as the reference allele in further analysis of the odds ratios. Tables 4 and 5 show the risk for lung cancer associated with each genotype (Table 4) or allele (Table 5) comparing frequencies of each genotype or allele in cases and controls with the most frequent genotype or allele as reference. In the Croatian subjects the cd genotype and in the Norwegian subjects the cc genotype was associated with a reduced risk of lung cancer, whereas combining both groups only cc genotype had a significant effect on reduction of the cancer risk (Table 4). Regarding allele frequencies, only subjects with a b allele in the Croatian population had a significant increased risk of lung cancer (Table 5).

**Table 2:** Genotypes frequencies of FAM46A VNTR polymorphisms in Croatian and Norwegian subjects with NSCLC and healthy controls.

Subjects	Variant		Genotype frequency	
Croatians	Genotype	VNTR	Patients (n = 262) frequency (N)	Controls (n = 568) frequency (N)
	Bb	3/3	24 (0.09)	36 (0.06)
	Cc	4/4	19 (0.07)	31 (0.05)
	dd	5/5	40 (0.15)	92 (0.16)
	ee	6/6	2 (0.01)	3 (0.01)
	ff	7/7	0	1 (0.00)
	ab	2/3	0	4 (0.01)
	ac	2/4	0	1 (0.00)
	ad	2/5	4 (0.02)	4 (0.01)
	ae	2/6	0	2 (0.00)
	af	2/7	0	1 (0.00)
	bc	3/4	38 (0.15)	63 (0.11)
	bd	3/5	69 (0.26)	135 (0.24)
	be	3/6	9 (0.03)	20 (0.04)
	bf	3/7	1 (0.00)	5 (0.01)
	cd	4/5	34 (0.13)	111 (0.20)
	ce	4/6	9 (0.03)	16 (0.03)
	cf	4/7	1 (0.00)	0
	de	5/6	12 (0.05)	39 (0.07)
	df	5/7	0	4 (0.01)
	ef	6/7	0	1 (0.00)
Norwegians	Genotype	VNTR	Patients (n = 241) frequency (N)	Controls (n = 329) frequency (N)
	bb	3/3	15 (0.06)	21 (0.06)
	cc	4/4	9 (0.04)	36 (0.12)
	dd	5/5	50 (0.21)	64 (0.20)
	ee	6/6	0	2 (0.01)
	ab	2/3	1 (0.001)	2 (0.01)
	ac	2/4	0	2 (0.01)
	ad	2/5	1 (0.001)	4 (0.01)
	ae	2/6	0	2 (0.01)
	bc	3/4	37 (0.15)	40 (0.12)
	bd	3/5	44 (0.18)	50 (0.15)
	be	3/6	12 (0.05)	10 (0.03)
	cd	4/5	47 (0.20)	56 (0.17)
	ce	4/6	7 (0.03)	11 (0.03)
	cf	4/7	1 (0.00)	1 (0.00)
	de	5/6	15 (0.62)	27 (0.08)
	df	5/7	1 (0.004)	1 (0.003)
	ef	6/7	1 (0.004)	

N: frequency of genotypes/alleles per group, n: total number of alleles per group.

**Table 3:** Alleles frequencies of FAM46A VNTR polymorphisms in Croatian and Norwegian subjects with NSCLC and healthy controls.

Subjects	Variant		Allele frequency	
Croatians	Allele	VNTR	Patients (n = 524) frequency (N)	Controls (n = 1136) frequency (N)
	a	2	4 (0.01)	11 (0.01)
	b	3	165 (0.31)	299 (0.26)
	c	4	120 (0.23)	253 (0.22)
	d	4	199 (0.38)	477 (0.42)
	e	6	34 (0.06)	84 (0.07)
	f	7	2 (0.004)	12 (0.01)
Norwegians	Allele	VNTR	Patients (n = 482) frequency (N)	Controls (n = 658) frequency (N)
	a	2	2 (0.004)	10 (0.02)
	b	3	124 (0.26)	144 (0.22)
	c	4	110 (0.23)	182 (0.28)
	d	4	208 (0.43)	266 (0.40)
	e	6	35 (0.07)	54 (0.08)
	f	7	3 (0.006)	2 (0.003)
Combined Croatians and Norwegians	Allele	VNTR	Patients (n = 1006) frequency (N)	Controls (n = 1794) frequency (N)
	a	2	6 (0.01)	21 (0.01)
	b	3	289 (0.29)	443 (0.25)
	c	4	230 (0.23)	435 (0.24)
	d	4	407 (0.40)	743 (0.41)
	e	6	69 (0.07)	138 (0.08)
	f	7	5 (0.005)	14 (0.01)

N: frequency of genotypes/alleles per group, n: total number of alleles per group

**Table 4:** Association of FAM46A VNTR genotypes with NSCLC in Croatian and Norwegian subjects.

Subjects	VNTR Variant	Unadjusted for covariates	Adjusted for covariates
Croatians	Genotype	OR(crude), 95% CI, pU N = 830	OR(adjusted), 95% CI, p^g^, N = 734
	bd	Reference	
	bb	1.03, 0.72–2.34, 0.38	1.26, 0.66–2.40, 0.48
	bc	1.18, 0.72–1.94, 0.51	1.09, 0.64–1.87, 0.76
	be	0.88, 0.38–2.04, 0.77	0.67, 0.24–1.93, 0.46
	cc	1.20, 0.63–2.28, 0.58	1.44, 0.72–2.86, 0.31
	cd	**0.60, 0.37–0.97, 0.037**	**0.56, 0.33–0.96, 0.035**
	ce	1.10, 0.43–2.62, 0.83	1.23, 0.49–3.12, 0.66
	dd	0.60, 0.30–1.22, 0.16	0.82, 0.5–1.37, 0.45
	de	0.80 0.51–1.25, 0.33	0.54, 2.5–1.21, 0.13
	LFG	0.51, 5.75E-06	0.55, 0.21–1.43, 0.22
Norwegians	Genotype	OR(crude), 95% CI, pU, N = 541	OR(adjusted), (95% CI), p^ags^, N = 538
	dd	Reference	
	bb	0.92, 0.42–1.99, 0.82	0.98, 0.44–2.18. 0.96
	bc	1.30, 0.71–2.34, 0.39	1.16, 0.62–2.18, 0.65
	bd	1.09, 0.62–1.9, 0.77	0.97, 0.54–1.74, 0.92
	be	1.39, 0.55–3.50, 0.48	1.29, 0.48–3.46, 0.62
	cc	**0.37, 0.16–0.86, 0.02**	**0.27, 0.11–0.69, 0.006**
	cd	1.11, 0.64–1.93, 0.70	1.05, 0.59–1.88, 0.86
	ce	0.81, 0.28–2.29, 0.69	0.77, 0.27–2.22, 0.63
	de	0.73, 0.34–1.53, 0.40	0.73, 0.33–1.59, 0.43
	LFG	1.08, 0.46–2.51. 0.86	1.14, 0.47–2.75, 0.78
Combined Croatians and Norwegians	Genotype	OR(crude), 95% CI, pU, N = 1371	OR(adjusted), 95% CI), p^ag^, N = 1222
	bd+dd	Reference	
	bb	1.16, 0.74–1.81, 0.51	1.27, 0.71–2.26, 0.43
	bc	1.28, 0.91–1.81, 0.17	1.25, 0.80–1.94, 0.33
	be	1.15, 0.64–2.05, 0.65	1.21, 0.55–2.67, 0.63
	cc	0.78, 0.48–1.26, 0.30	**0.55, 0.30–0.996, 0.048**
	cd	0.53, 0.60–1.14, 0.25	0.90, 0.60–1.35, 0.61
	ce	1.01, 0.53–1.92, 0.98	0.97, 0.43–2.14, 0.91
	de	0.70, 0.43–1.14, 0.15	0.68, 0.37–1.22, 0.19
	LFG	0.88, 0.50–1.54. 0.66	1.05, 0.52–2.12, 0.90

p^U^: unadjusted p-value,

p^g^: p-value adjusted for gender,

p^ag^: p-value adjusted for age and gender,

p^ags^: p-value adjusted for age, gender and smoking status,

N: number of alleles or genotypes include in the analysis, LFG: Low frequency genotypes (ab+ac+ad+ae+bf+cf+af+ee+ef+ff), CI: confidence interval, OR: Odds ratio

**Table 5:** Association of FAM46A VNTR alleles with NSCLC in Croatian and Norwegian subjects.

Subjects		VNTR Variant		Unadjusted for covariates		Adjusted for covariates	
Croatians		Allele		OR(crude), 95% CI, pU, N = 1660		OR(adjusted), 95% CI, p^g^, N = 1468	
		d		Reference			
		a		0.87, 0.27–2.77, 0.82		0.85, 0.23–3.15, 0.81	
		b		**1.32, 1.03–1.70, 0.03**		**1.32, 1.003–1.74, 0.048**	
		c		1.14, 0.87–1.49, 0.36		1.18, 0.88–1.59, 0.27	
		e		0.97, 0.63–1.49, 0.89		0.89, 0.54–1.46, 0.64	
		f		0.40, 0.09–1.80, 0.23		0.44, 0.10–2.02, 0.29	
Norwegians		Allele		OR(crude), 95% CI, pU, N = 1162		OR, 95% CI, p^ags^, N = 1075	
		d		Reference			
		a		0.25, 0.06–1.17, 0.08		0.32, 0.07–1.52, 0.15	
		b		1.09, 0.81–1.47, 0.58		1.07, 0.78–1.48, 0.68	
		c		0.76, 0.56–1.02, 0.07		0.75,0.54–1.04, 0.08	
		e		0.82, 0.52–1.30, 0.40		0.80, 0.49–1.31, 0.37	
		f		1.90, 0.31–11.44, 0.49		1.89, 0.31–11.76, 0.49	
Combined Croatians and Norwegians		Allele		OR(crude), 95% CI, pU, N = 2800		OR(adjusted), 95% CI, p^ag^, N = 2444	
		d		Reference			
		a		0.52, 0.21–1.30, 0.16		0.53, 0.17–1.68, 0.28	
		b		1.19, 0.98–1.44, 0.07		1.20, 0.94–1.53, 0.15	
		c		0.97, 0.79–1.18, 0.76		0.91, 0.71–1.18, 0.49	
		e		0.90), 0.66–1.23, 0.51		0.84, 0.56–1.25, 0.39	
		f		0.65, 0.23–1.82, 0.42		1.21, 0.35–4.15, 0.76	

p^U^: unadjusted p-value,

p^g^: p-value adjusted for gender,

p^ag^: p-value adjusted for age and gender,

p^ags^: p-value adjusted for age, gender and smoking status,

N: number of alleles or genotypes include in the analysis, CI: confidence interval, OR: Odds ratio.

### Genotypic and Allelic frequencies of BAG6 rs3117582 SNP

For the *BAG6* rs3117582 polymorphism we found a gene-dosage increased risk for lung cancer associated with the C allele of *BAG6* rs3117582 SNP in the Norwegian subjects. The subjects carrying one C allele had 1.70-fold increased lung cancer risk and subjects with two variant alleles (CC) had almost 7-fold increased risk of lung cancer. No such association was present in the Croatian subjects; however, combining the subjects from both populations the associations remained significant and similar to the associations found in the Norwegian population (Table 6).

**Table 6:** Association of BAG6 SNP 3117582 with NSCLC in Croatian and Norwegian subjects.

Subjects	SNP Variant	Number of cases (n)	Number of healthy controls (n)	Unadjusted for covariates	Adjusted for covariates
Croatians	Genotype	n = 262	n = 568	OR(crude), 95% CI, p^U^	OR(adjusted), 95% CI, p^ag^
	AA	218	491	Reference	
	AC	41	69	1.34, 0.88–2.03, 0.17	1.46, 0.94–2.29, 0.095
	CC	2	4	1.13, 0.21–6.19, 0.89	1.59, 0.26–6.67, 0.61
Norwegians	Genotype	n = 249	n = 332	OR(crude), 95% CI, p^U^	OR(adjusted), 95% CI, p^ags^
	AA	170	271	Reference	
	AC	62	58	**1.70, 1.16–2.56, 0.01**	**1.68, 1.08–2.61, 0.02**
	CC	8	3	**4.25, 1.11–16–25, 0.03**	**7.06, 1.45–34.40, 0.016**
Combined Croatians and Norwegians	Genotype	n = 498	n = 664	OR(crude), 95% CI, p^U^	OR (adjusted), 95% CI, p^ag^
	AA	340	542	Reference	
	AC	124	116	**1.70, 1.28–2.27, 0.0003**	**1.72, 1.27–2.33, 0.0005**
	CC	16	6	**4.25, 1.65–10.97, 0.003**	**6.89, 2.24–21.14, 0.0007**
Croatians	Allele	n = 522	n = 1128	OR(crude), 95% CI, p^U^	OR(adjusted), 95% CI, p^ag^
	a	475	1050	Reference	
	c	47	78	1.33, 0.91–1.94, 0.14	1.48, 0.99–2.22, 0.056
Norwegians	Allele	498	n = 664	OR(crude), 95% CI, p^U^	OR(adjusted), 95% CI, p^ags^
	a	340	542	Reference	
	c	140	122	**1.83, 1.39–2.42, 0.00002**	**1.88, 1.39–2.54, 0.00004**
Combined Croatians and Norwegians	Allele	n = 1022	n = 1800	OR(crude), 95% CI, p^U^	OR(adjusted), 95% CI, p^ag^
	a	815	1529	Reference	
	c	187	200	**1.83, 1.47–2,67, 4.96x10** ^**-8**^	**1.63, 1.24–2.13, 0.0004**

p^U^: unadjusted p-value,

p^g^: p-value adjusted for gender,

p^ag^: p-value adjusted for age and gender,

p^ags^: p-value adjusted for age, gender and smoking status,

N: number of alleles or genotypes include in the analysis, n: numbers of alleles or genotypes, CI: confidence interval, OR: Odds ratio.

## Discussion

The present study investigates the association between a VNTR in exon 2 of the *FAM46A* gene and a SNP (rs3117582) in the *BAG6* gene and risk of NSCLC in two case-control studies from Croatia and Norway. We found that an allele of *FAM46A* gene that carries three VNTR repeats (designated as allele b) was associated with increased risk of lung cancer in the Croatian subjects. In addition, a genotype of *FAM46A* gene with 4 and 5 VNTR repeats (designated cd) was associated with reduced risk of cancer in these subjects. However, another genotype (cc) conferred reduced risk to lung cancer in the Norwegian subjects. To date, this is the first report of an association between the *FAM46A* gene and NSCLC

Of particular interest is our observation that the cc and cd genotypes of the *FAM46A* gene confer reduced risk to NSCLC in the Norwegian and Croatian subjects, respectively. In addition, we found that the *FAM46A* bd and dd genotypes were the dominant genotypes in the Croatian and Norwegian subjects, respectively. These findings may suggest that the frequency of the dominant genotype of the *FAM46A* gene may influence the particular genotypes(s) that associates with NSCLC risk in each population. There is the possibility that these polymorphisms could be markers for susceptibility or reduced risk factor for example binding sites for miRNA, CpG methylation changes, SNPs in vicinity or genetic instability. Our results may therefore encourage further studies aimed at replicating our results, validating and/or eliminating the possibility of false positives or negatives that may be present in our results as a consequence of these genetic events.

The function of the *FAM46A* gene and its component domains (PS50315 and DUF1693) is unknown [25]. The DUF domain is present in many hypothetical proteins including nematode prion-like proteins [27] and in nucleotidyltransferase superfamily genes with unknown function [27]. Our results suggest that the VNTR-encoded PS50315 domain of the *FAM46A* protein might have functional importance in NSCLC. The *FAM46A* gene might have a role in *TGF-β* signaling, cell death (apoptosis) and/or inflammation due to its interaction with the *ZFYVE9* protein [28]. An experimentally determined interacting partner of the *FAM46A* protein is the *ZFYVE9* protein [28]. *ZFYVE9* protein is involved in the recruitment of unphosphorylated forms of *SMAD2/SMAD3* to the *TGF-*β receptor (*TGF-βR*) [39]. Phosphorylation of *SMAD2/SMAD3* induces dissociation from *ZFYVE9*. This allows for the formation of SMAD2/SMAD4 complexes and the consequent translocation of the *SMAD2/SMAD4* complexes to the nucleus. Perhaps the *FAM46A* protein is involved in this cascade of events. *TGF-β* is a potent inhibitor of cell growth and accumulating evidence suggests that perturbation of *TGF-β* signaling pathway leads to tumorigenesis [40]. It is therefore tempting to speculate that *FAM46A* protein may be involved in lung cancer etiology through its participation in the *TGF-β* signaling pathway. This may involve the VNTR-encoded PS50315 domain of *FAM46A* gene. Our results may therefore encourage further studies to validate this postulation.

We found that the *BAG6* rs3117582 SNP is associated with NSCLC both at the genotypic and allelic levels in the Norwegian subjects and the combined Croatian and Norwegian subjects. This is interesting since *BAG6* protein is an interacting partner of *FAM46A* protein [28]. Previous reports have shown that SNPs in *BAG6* gene (rs1052486 and rs3117582) conferred susceptibility to lung cancer [8;30]. Our results support these previous reports and further suggest a role for the *BAG6* gene in the etiology of non small cell lung cancer. *BAG6* polypeptides regulates a variety of cellular processes such as apoptosis [41], HLA class II expression [42], T cell responses [43], protein modification and gene expression [44]. All these processes may require that the *BAG6* protein is constitutively expressed at steady-state levels for optimal functioning. In addition, the *BAG6* rs3117582 SNP is located at the promoter region of the *BAG6* gene (38 basepairs from the transcription start site). Mutations at the promoter region of genes have been shown to influence the expression levels of the respective genes. This may concurrently attenuate the physiological efficiency of the genes in a dose-response manner [45;46]. Our results suggest that the C allele of *BAG6* rs3117582 SNP is associated with increased risk for NSCLC in the Norwegian subjects and the combined Croatian and Norwegian subjects in a gene-dosage manner. This may also suggest that, the presence of the minor C allele of *BAG6* rs3117582 SNP in its promoter may perturbed the *BAG6* gene expression. This perturbation may lead to significant increase in NSCLC risk in individuals that bear this allele. This postulation needs to be investigated in further studies to determine and ascertain the possible underlying mechanism(s) of action.

In conclusion, our study suggests that the *FAM46A* gene VNTR and *BAG6* rs3117582 SNP are associated with NSCLC in both the Croatian and Norwegian populations. Our results also corroborate the previous findings that *BAG6* gene is associated with lung cancer and suggest gene-dosage association of the BAG rs3117582 SNP with NSCLC. We further suggest that these loci could be potential research targets that might have therapeutic potentials.

## References

[1] Jemal A, Bray F, Center MM, Ferlay J, Ward E, Forman D. Global cancer statistics. CA Cancer J Clin 2011;61:69–90. 10.3322/caac.20107 21296855

[2] Spitz MR, Hong WK, Amos CI, Wu X, Schabath MB, Dong Q, et al A risk model for prediction of lung cancer. J Natl Cancer Inst 2007;99:715–726. 17470739 10.1093/jnci/djk153

[3] Albright F, Teerlink C, Werner TL, Cannon-Albright LA. Significant evidence for a heritable contribution to cancer predisposition: a review of cancer familiality by site. BMC Cancer 2012;12:138 10.1186/1471-2407-12-138 22471249 PMC3350420

[4] Brennan P, Hainaut P, Boffetta P. Genetics of lung-cancer susceptibility. Lancet Oncol 2011;12:399–408. 10.1016/S1470-2045(10)70126-1 20951091

[5] Cote ML, Liu M, Bonassi S, Neri M, Schwartz AG, Christiani DC, et al Increased risk of lung cancer in individuals with a family history of the disease: a pooled analysis from the International Lung Cancer Consortium. Eur J Cancer 2012;48:1957–1968. 10.1016/j.ejca.2012.01.038 22436981 PMC3445438

[6] Dong J, Jin G, Wu C, Guo H, Zhou B, Lv J, et al Genome-wide association study identifies a novel susceptibility locus at 12q23.1 for lung squamous cell carcinoma in han chinese. PLoS Genet 2013;9:e1003190 10.1371/journal.pgen.1003190 23341777 PMC3547794

[7] Hart K, Landvik NE, Lind H, Skaug V, Haugen A, Zienolddiny S. A combination of functional polymorphisms in the CASP8, MMP1, IL10 and SEPS1 genes affects risk of non-small cell lung cancer. Lung Cancer 2011;71:123–129. 10.1016/j.lungcan.2010.04.016 20471133

[8] Rudd MF, Webb EL, Matakidou A, Sellick GS, Williams RD, Bridle H, et al Variants in the GH-IGF axis confer susceptibility to lung cancer. Genome Res 2006;16:693–701. 16741161 10.1101/gr.5120106 PMC1473180

[9] Timofeeva MN, Hung RJ, Rafnar T, Christiani DC, Field JK, Bickeboller H, et al Influence of common genetic variation on lung cancer risk: meta-analysis of 14 900 cases and 29 485 controls. Hum Mol Genet 2012;21:4980–4995. 10.1093/hmg/dds334 22899653 PMC3607485

[10] Truong T, Sauter W, McKay JD, Hosgood HD III, Gallagher C, Amos CI, et al coordinated association study of 10 potential lung cancer susceptibility variants. Carcinogenesis 2010;31:625–633. 10.1093/carcin/bgq001 20106900 PMC2847090

[11] Walsh KM, Gorlov IP, Hansen HM, Wu X, Spitz MR, Zhang H, et al Fine-mapping of the 5p15.33, 6p22.1-p21.31, and 15q25.1 regions identifies functional and histology-specific lung cancer susceptibility loci in African-Americans. Cancer Epidemiol Biomarkers Prev 2013;22:251–260. 10.1158/1055-9965.EPI-12-1007-T 23221128 PMC3565099

[12] Azzoli CG, Park BJ, Pao W, Zakowski M, Kris MG. Molecularly tailored adjuvant chemotherapy for resected non-small cell lung cancer: a time for excitement and equipoise. J Thorac Oncol 2008;3:84–93. 10.1097/JTO.0b013e31815efe24 18166846

[13] Jacobsen B, Kriegbaum MC, Santoni-Rugiu E, Ploug M. C4.4A as a biomarker in pulmonary adenocarcinoma and squamous cell carcinoma. World J Clin Oncol 2014;5:621–632. 10.5306/wjco.v5.i4.621 25302166 PMC4129527

[14] Farhat FS, Tfayli A, Fakhruddin N, Mahfouz R, Otrock ZK, Alameddine RS, et al Expression, prognostic and predictive impact of VEGF and bFGF in non-small cell lung cancer. Crit Rev Oncol Hematol 2012;84:149–160. 10.1016/j.critrevonc.2012.02.012 22494932

[15] Campbell L, Blackhall F, Thatcher N. Gefitinib for the treatment of non-small-cell lung cancer. Expert Opin Pharmacother 2010;11:1343–1357. 10.1517/14656566.2010.481283 20426712

[16] Gridelli C, Maione P, Bareschino MA, Schettino C, Sacco PC, Ambrosio R, et.al Erlotinib in the treatment of non-small cell lung cancer: current status and future developments. Anticancer Res 2010;30:1301–1310. 20530444

[17] Hannan AJ. TRPing up the genome: Tandem repeat polymorphisms as dynamic sources of genetic variability in health and disease. Discov Med 2010;10:314–321. 21034672

[18] Hannan AJ. Tandem repeat polymorphisms: modulators of disease susceptibility and candidates for 'missing heritability'. Trends Genet 2010;26:59–65. 10.1016/j.tig.2009.11.008 20036436

[19] Lindstedt BA, Ryberg D, Zienolddiny S, Khan H, Haugen A. Hras1 VNTR alleles as susceptibility markers for lung cancer: relationship to microsatellite instability in tumors. Anticancer Res 1999;19:5523–5527. 10697610

[20] Ryberg D, Lindstedt BA, Zienolddiny S, Haugen A. A hereditary genetic marker closely associated with microsatellite instability in lung cancer. Cancer Res 1995;55:3996–3999. 7664270

[21] Hu Z, Shao M, Chen Y, Zhou J, Qian J, Xu L, et al Allele 2 of the interleukin-1 receptor antagonist gene (IL1RN*2) is associated with a decreased risk of primary lung cancer. Cancer Lett 2006;236:269–275. 16019127 10.1016/j.canlet.2005.05.015

[22] Lind H, Zienolddiny S, Ryberg D, Skaug V, Phillips DH, Haugen A. Interleukin 1 receptor antagonist gene polymorphism and risk of lung cancer: a possible interaction with polymorphisms in the interleukin 1 beta gene. Lung Cancer 2005;50:285–290. 16126303 10.1016/j.lungcan.2005.07.003

[23] Liu B, Yang L, Huang B, Cheng M, Wang H, Li Y, et al A functional copy-number variation in MAPKAPK2 predicts risk and prognosis of lung cancer. Am J Hum Genet 2012;91:384–390. 10.1016/j.ajhg.2012.07.003 22883146 PMC3415537

[24] Lagali PS, Kakuk LE, Griesinger IB, Wong PW, Ayyagari R. Identification and characterization of C6orf37, a novel candidate human retinal disease gene on chromosome 6q14. Biochem Biophys Res Commun 2002;293:356–365. 12054608 10.1016/S0006-291X(02)00228-0

[25] Barragan I, Borrego S, Abd El-Aziz MM, El-Ashry MF, Abu-Safieh L, Bhattacharya SS, et al Genetic analysis of FAM46A in Spanish families with autosomal recessive retinitis pigmentosa: characterisation of novel VNTRs. Ann Hum Genet 2008;72:26–34. 17803723 10.1111/j.1469-1809.2007.00393.x

[26] Cui J, Wang W, Lai MD, Xu EP, Lv BJ, Lin J, et al Identification of a novel VNTR polymorphism in C6orf37 and its association with colorectal cancer risk in Chinese population. Clin Chim Acta 2006;368:155–159. 16545789 10.1016/j.cca.2005.12.043

[27] Kuchta K, Knizewski L, Wyrwicz LS, Rychlewski L, Ginalski K. Comprehensive classification of nucleotidyltransferase fold proteins: identification of novel families and their representatives in human. Nucleic Acids Res 2009;37:7701–7714. 10.1093/nar/gkp854 19833706 PMC2794190

[28] Colland F, Jacq X, Trouplin V, Mougin C, Groizeleau C, Hamburger A, et al Functional proteomics mapping of a human signaling pathway. Genome Res 2004;14:1324–1332. 15231748 10.1101/gr.2334104 PMC442148

[29] Lim J, Hao T, Shaw C, Patel AJ, Szabo G, Rual JF, et al A protein-protein interaction network for human inherited ataxias and disorders of Purkinje cell degeneration. Cell 2006;125:801–814. 16713569 10.1016/j.cell.2006.03.032

[30] Wang Y, Broderick P, Webb E, Wu X, Vijayakrishnan J, Matakidou A, et al Common 5p15.33 and 6p21.33 variants influence lung cancer risk. Nat Genet 2008;40:1407–1409. 10.1038/ng.273 18978787 PMC2695928

[31] Wu YH, Shih SF, Lin JY. Ricin triggers apoptotic morphological changes through caspase-3 cleavage of BAT3. J Biol Chem 2004;279:19264–19275. 14960581 10.1074/jbc.M307049200

[32] Etokebe GE, Kuchler AM, Haraldsen G, Landin M, Osmundsen H, Dembic Z. Family-with-sequence-similarity-46, member A (Fam46a) gene is expressed in developing tooth buds. Arch Oral Biol 2009;54:1002–1007. 10.1016/j.archoralbio.2009.08.005 19740458

[33] Etokebe GE, Jotanovic Z, Mihelic R, Jericevic BM, Nikolic T, Balen S, et al. Susceptibility to large-joint osteoarthritis (hip and knee) is associated with BAG6 rs3117582 SNP and the VNTR polymorphism in the second exon of the FAM46A gene on chromosome 6. J Orthop Res 2014. 10.1002/jor.22738 25231575

[34] Etokebe GE, Bulat-Kardum L, Munthe LA, Balen S, Dembic Z. Association of variable number of tandem repeats in the coding region of the FAM46A gene, FAM46A rs11040 SNP and BAG6 rs3117582 SNP with susceptibility to tuberculosis. PLoS One 2014;9:e91385 10.1371/journal.pone.0091385 24625963 PMC3953334

[35] Fan WC, Ting WY, Lee MC, Huang SF, Chiu CH, Lai SL, et al Latent TB infection in newly diagnosed lung cancer patients—A multicenter prospective observational study. Lung Cancer 2014;85:472–478. 10.1016/j.lungcan.2014.07.001 25063540

[36] Jotanovic Z, Etokebe GE, Mihelic R, Heiland KM, Mulac-Jericevic B, Tijanic T, et al Hip osteoarthritis susceptibility is associated with IL1B -511(G>A) and IL1 RN (VNTR) genotypic polymorphisms in Croatian Caucasian population. J Orthop Res 2011;29:1137–1144. 10.1002/jor.21378 21671260

[37] Jotanovic Z, Etokebe GE, Mihelic R, Kaarvatn MH, Mulac-Jericevic B, Tijanic T, et al IL1B -511(G>A) and IL1RN (VNTR) allelic polymorphisms and susceptibility to knee osteoarthritis in Croatian population. Rheumatol Int 2012;32:2135–2141. 10.1007/s00296-011-1946-3 21523343

[38] Kaarvatn MH, Jotanovic Z, Mihelic R, Etokebe GE, Mulac-Jericevic B, Tijanic T, et al Associations of the interleukin-1 gene locus polymorphisms with risk to hip and knee osteoarthritis: gender and subpopulation differences. Scand J Immunol 2013;77:151–161. 10.1111/sji.12016 23216199

[39] Tsukazaki T, Chiang TA, Davison AF, Attisano L, Wrana JL. SARA, a FYVE domain protein that recruits Smad2 to the TGFbeta receptor. Cell 1998;95:779–791. 9865696 10.1016/s0092-8674(00)81701-8

[40] Kawabata M, Imamura T, Inoue H, Hanai J, Nishihara A, Hanyu A, et al Intracellular signaling of the TGF-beta superfamily by Smad proteins. Ann N Y Acad Sci 1999;886:73–82. 10667205 10.1111/j.1749-6632.1999.tb09402.x

[41] Sasaki T, Gan EC, Wakeham A, Kornbluth S, Mak TW, Okada H. HLA-B-associated transcript 3 (Bat3)/Scythe is essential for p300-mediated acetylation of p53. Genes Dev 2007;21:848–861. 17403783 10.1101/gad.1534107 PMC1838535

[42] Kamper N, Franken S, Temme S, Koch S, Bieber T, Koch N. gamma-Interferon-regulated chaperone governs human lymphocyte antigen class II expression. FASEB J 2012;26:104–116. 10.1096/fj.11-189670 21940994

[43] Rangachari M, Zhu C, Sakuishi K, Xiao S, Karman J, Chen A, et al Bat3 promotes T cell responses and autoimmunity by repressing Tim-3-mediated cell death and exhaustion. Nat Med 2012;18:1394–1400. 22863785 10.1038/nm.2871 PMC3491118

[44] Nguyen P, Bar-Sela G, Sun L, Bisht KS, Cui H, Kohn E, et al BAT3 and SET1A form a complex with CTCFL/BORIS to modulate H3K4 histone dimethylation and gene expression. Mol Cell Biol 2008;28:6720–6729. 10.1128/MCB.00568-08 18765639 PMC2573239

[45] Aguillon JC, Cruzat A, Aravena O, Salazar L, Llanos C, Cuchacovich M. Could single-nucleotide polymorphisms (SNPs) affecting the tumour necrosis factor promoter be considered as part of rheumatoid arthritis evolution? Immunobiology 2006;211:75–84. 16446172 10.1016/j.imbio.2005.09.005

[46] Shih MC, Chiu YN, Hu MC, Guo IC, Chung BC. Regulation of steroid production: analysis of Cyp11a1 promoter. Mol Cell Endocrinol 2011;336:80–84. 10.1016/j.mce.2010.12.017 21195129

